# Hand and finger, ultrasound‐guided, percutaneous core needle biopsies: A safe procedure with high diagnostic accuracy

**DOI:** 10.1002/ajum.12365

**Published:** 2023-10-24

**Authors:** Stephanie Magoon, Vanessa Peters, Felipe Ferreira de Souza, David Chen, Patrick Owens, Juan Pretell‐Mazzini, Natalia Fullerton, Jean Jose, Andrew Rosenberg, Ty K. Subhawong

**Affiliations:** ^1^ Leonard M. Miller School of Medicine, University of Miami Miami Florida USA; ^2^ Department of Radiology University of Miami Miller School of Medicine and the Sylvester Comprehensive Care Center Miami Florida USA; ^3^ Department of Orthopedic Surgery University of Miami Miller School of Medicine and the Sylvester Comprehensive Care Center Miami Florida USA; ^4^ Department of Orthopedics Herbert Wertheim College of Medicine, Florida International University Miami Florida USA; ^5^ Department of Plastic Surgery University of Miami Miller School of Medicine and the Sylvester Comprehensive Care Center Miami Florida USA; ^6^ Department of Pathology University of Miami Miller School of Medicine and the Sylvester Comprehensive Care Center Miami Florida USA

## Abstract

**Introduction/Purpose:**

To determine the diagnostic accuracy and complication rates of ultrasound‐guided, percutaneous core needle biopsies of soft tissue masses in the hand and fingers.

**Methods:**

Reports from all ultrasound‐guided procedures between 21 May 2014 and 17 March 2022 were queried for keywords including “hand”, OR “finger”, AND “biopsy”. Patient demographics, lesion size and location, biopsy needle gauge and the number of cores obtained were recorded. The final pathology of the mass excision was then compared with the core needle biopsy (CNB) for each patient.

**Results:**

Sixty‐six records were reviewed, and 37 patients met inclusion criteria. Maximum lesion diameter averaged 1.45 cm with a range between 0.4 and 4.3 cm. The frequency of needle gauges used was 14G (14%), 16G (24%), 18G (38%), 20G (11%) and ‘not reported’ (14%). The mean number of tissue cores obtained was 2.9 (SD 1.2; range 1 to 6), excluding nine cases that reported ‘multiple’. The frequency of CNB diagnoses included tenosynovial giant cell tumour (TGCT) at 30%, ganglion cyst at 11% and epidermal inclusion cyst at 5%. CNB was 100% sensitive in detecting the three (8%) malignancies. Of the 37 tumours biopsied, 16 were surgically excised. One angiomyoma was originally diagnosed as a haemangioma on CNB, but all other histologic results were concordant for a diagnostic accuracy of 97%.

**Discussion:**

Small soft tissue masses in the hands and fingers, even those less than 1 cm, are often amenable to ultrasound‐guided CNB. Performance under image guidance facilitates retrieval of core specimens adquate for histologic diagnosis with relatively few passes using higher gauge needles.

**Conclusion:**

Overall, ultrasound‐guided CNB of the hand and fingers is safe and highly accurate in diagnosing soft tissue tumours. The accuracy is unrelated to the needle's gauge, the number of passes and the size of the lesions.

## Introduction

Confirming diagnosis histologically in potentially malignant soft tissue masses dictates urgency and modality of treatment, patient response and prognosis. Fifteen per cent of soft tissue tumours are found in the hand.^1^, ^2^ While most soft tissue masses of the hand are benign,^1^ clinicians should have a low threshold for recruiting advanced diagnostic techniques as history and mass characteristics alone are insufficient to rule out malignancy in all patients. Biopsy of indeterminate soft tissue masses allows for definitive treatment planning and mitigates perioperative uncertainty.

While open biopsy techniques have traditionally been used for tissue sampling of the hand, they are associated with increased risk of complications including wound infection, haematoma and tumour seeding that can interfere with subsequent treatment planning.^3^, ^4^ Open biopsy increases utilisation of limited resources such as operating room time and increases costs. Ultrasound‐guided core needle biopsy (CNB) is a cost‐effective minimally invasive technique enabling precisely targeted tissue sampling, may be performed in the outpatient setting and is associated with a limited area of contamination.^3^, ^4^

Hesitancy in performing ultrasound‐guided biopsy of hand or finger masses likely arises from uncertainty about diagnostic yield and safety. Many studies describe the diagnostic value of ultrasound‐guided CNB in soft tissue masses of the extremities;^5^, ^6^ however, there are limited data on the diagnostic yield, accuracy and safety of this procedure specifically in the hand and fingers. Masses distal to the radiocarpal joint are often subcutaneous and evolve within the confined compartment of the hand. Conspicuity of these lesions and mass effect on local structures, such as tendons, nerves and vessels, leads to early detection of smaller masses and increased technical difficulty in obtaining diagnostic tissue percutaneously.^1^, ^4^ Given their unique clinical and anatomic considerations, we aimed to determine the diagnostic accuracy and complication rates of ultrasound‐guided CNB of soft tissue masses in the hand and fingers.

## Materials and methods

In this single‐institution, IRB‐approved retrospective study, the requirement for informed consent was waived (ID: 20190880). Radiology reports from all ultrasound‐guided procedures between 21 May 2014 and 17 March 2022 were queried for keywords including “hand”, OR “finger”, AND “biopsy”. Only soft tissue tumours were included. We excluded bone biopsies, cases where only fine‐needle aspiration or cyst aspiration was performed, and those that were proximal to the radiocarpal joint. Patient demographics including age at time of biopsy and gender were recorded. Lesion maximum diameter, lesion location, biopsy needle gauge and the number of cores obtained were recorded.

The biopsy needles used included 14‐, 16‐, 18‐ and 20‐gauge Temno™ (Merit Medical Systems, South Jordan, UT, USA) and SuperCore™ (Argon Medical Devices, Plano, TX, USA) devices, which are of similar design and employ a 1‐ or 2‐cm specimen notch and spring‐loaded cutting cannula. The biopsy needle gauge and number of cores sampled were determined by the radiologist performing the procedure and varied according to lesion‐specific clinical circumstances. There were three MSK fellowship‐trained radiologists involved in performing all of the ultrasound‐guided core needle biopsies (TKS, JJ and FS). They each had 10, 14 and 11 years of subspecialty MSK radiology experience, respectively, up until the date of the last biopsy in 2022. The final pathology of the excised mass (if obtained) was then compared with the CNB histology results. Time interval between the CNB and surgery was also noted, as were any complications that occurred within 30 days of CNB. The Society of Interventional Radiology (SIR) guidelines were used as a standard grading system for complication severity.

## Results

**Figure 1:**
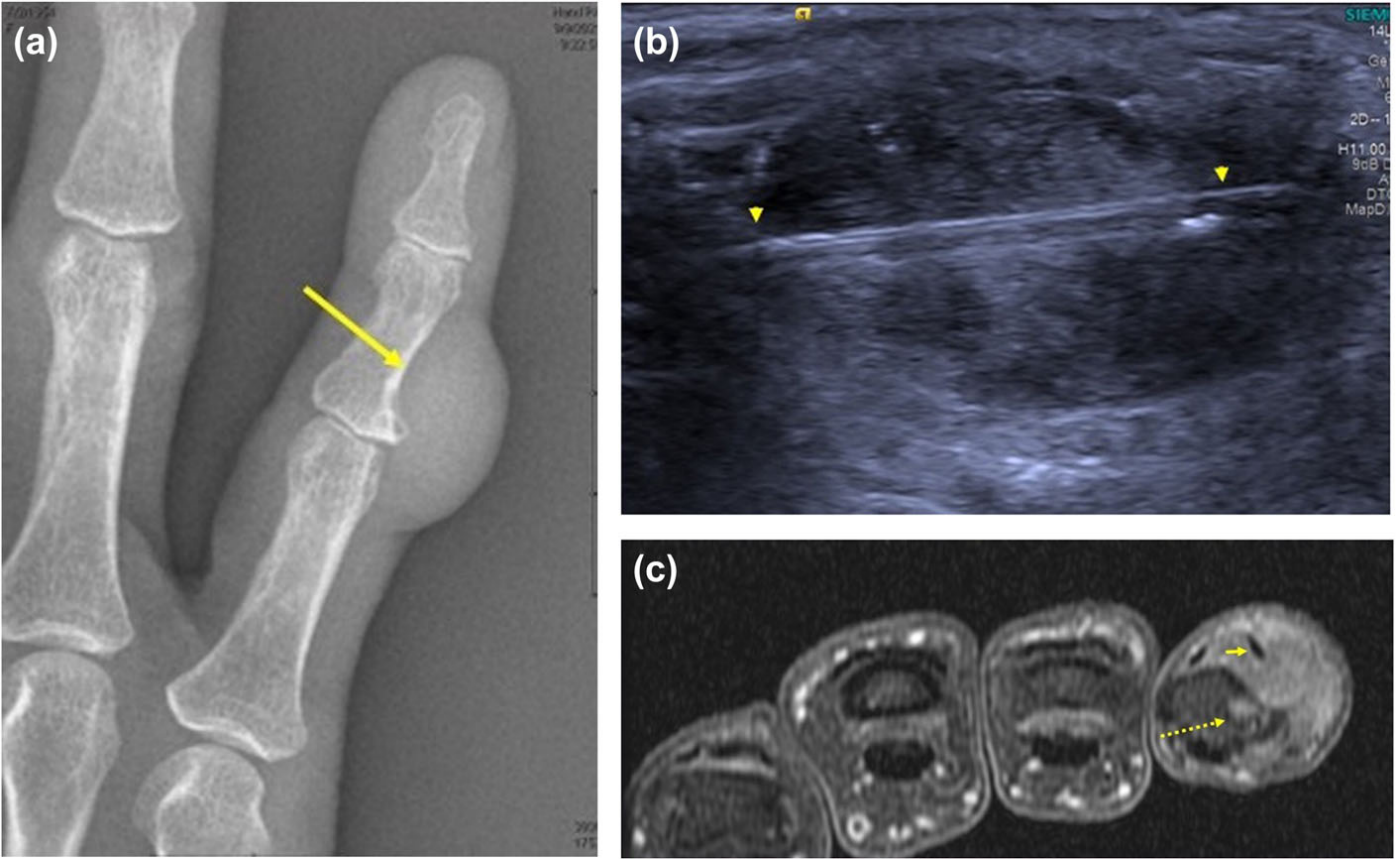
A 67‐year‐old woman with small finger soft tissue mass. (a) Radiograph demonstrates a soft tissue mass arising from the dorsal and ulnar aspect of the small finger, scalloping the middle phalanx cortex (arrow). (b) Axial, contrast‐enhanced, fat‐suppressed, T1‐weighted MRI shows a hypoenhancing soft tissue mass partially encompassing the middle phalanx, completely encasing and displacing the ulnar‐sided lateral band of the extensor tendon (short arrow) and eroding bone (dashed arrow). (c) Ultrasound‐guided biopsy with an 18‐gauge needle *via* longitudinal approach allowed adequate specimen retrieval to confirm tenosynovial giant cell tumour.

**Figure 2:**
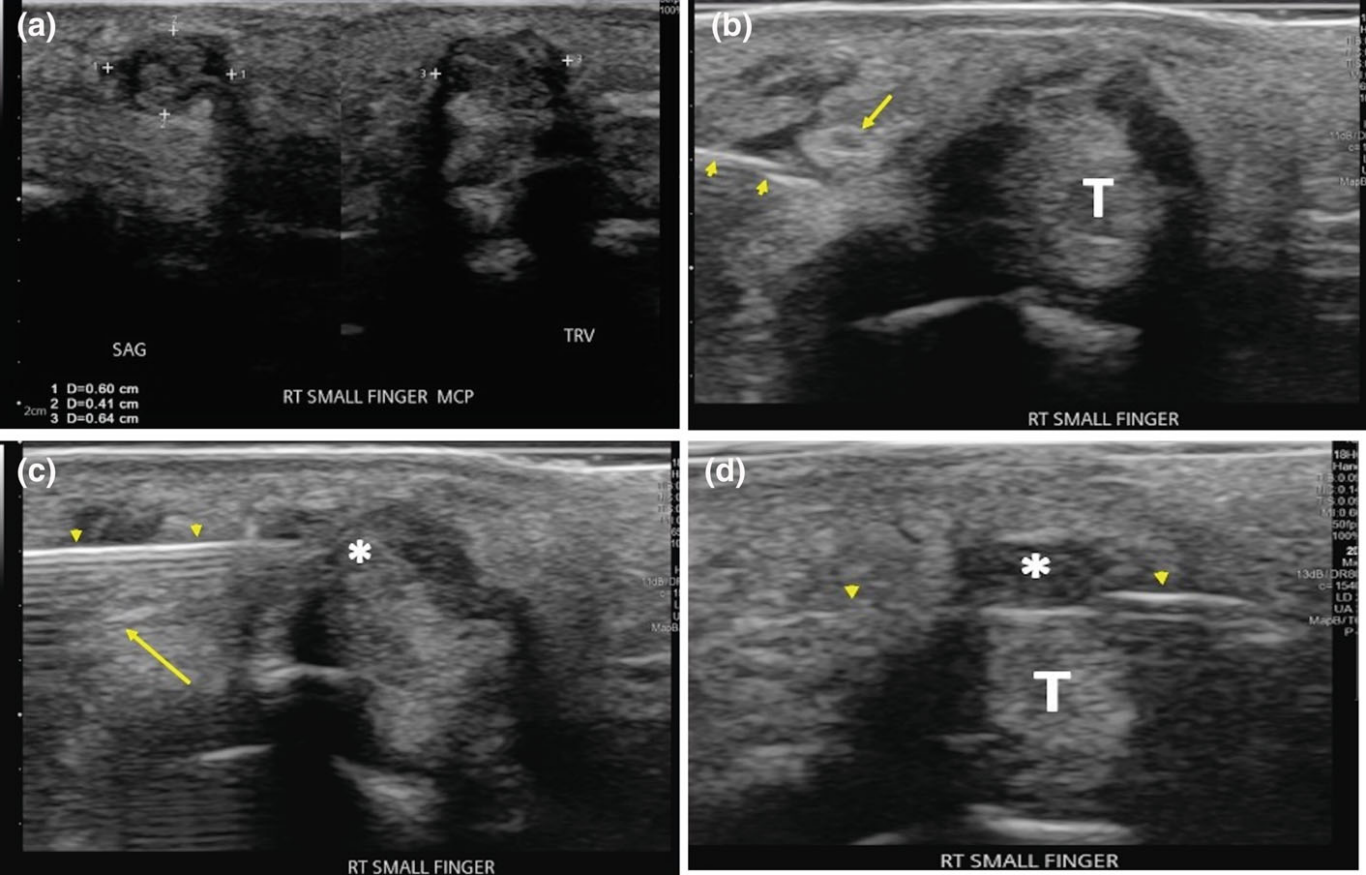
A 51‐year‐old woman with a palpable nodule over the volar aspect of small finger. (a) Sagittal and transverse ultrasound demonstrates a subcentimetre complex‐appearing nodule with internal echogenicity, abutting the flexor tendon sheath. (b) Transverse ultrasound shows a medial approach using a 22‐gauge hypodermic needle (arrowheads) to block the ulnar digital nerve (arrow) with 2% lidocaine; small finger flexor tendon (T). (c) The 18‐gauge needle (arrowheads) was carefully angled, superficial to the digital nerve (arrow) and tangential to tendon sheath, targeting the subcentimetre nodule (*). (d) 18‐gauge biopsy needle with open chamber (arrowheads) through the tendon sheath nodule (*), superficial to the flexor tendon (T). Note that the bevel tip was passed completely through the nodule to maximise the length of the retrieved tissue in the specimen chamber. Ultrasound‐guided biopsy with an 18‐gauge needle confirmed ganglion cyst.

**Figure 3:**
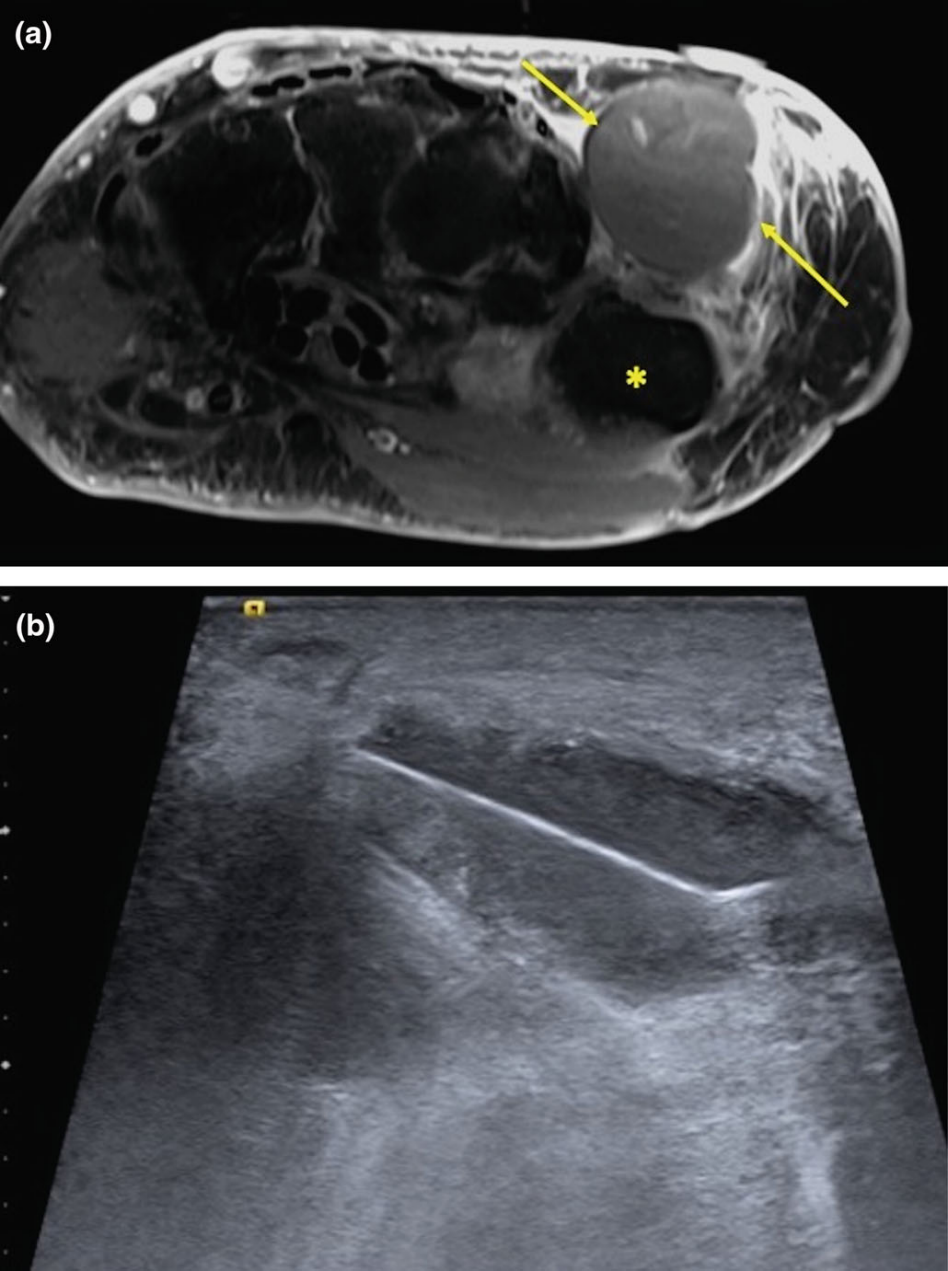
A 67‐year‐old man with a history of previously resected mass from first web space. (a) Contrast‐enhanced, fat‐suppressed, T1‐weighted MRI shows a non‐enhancing cystic mass (arrows) in the first web space, dorsal to the thumb metacarpal (*), and surrounding soft tissue enhancement. (b) Ultrasound‐guided biopsy with a 16‐gauge needle confirmed recurrent carcinosarcoma.

Sixty‐six patient records were reviewed, and 37 patients met inclusion criteria. The mean age was 55, with a range between 19 and 78, 65% of whom were females. Maximum lesion diameter averaged 1.45 cm with a range between 0.4 and 4.3 cm. The frequency of needle gauges used was 14G (14%), 16G (24%), 18G (38%), 20G (11%) and ‘not reported’ (14%). The mean number of tissue cores obtained was 2.9 (SD 1.2; range 1–6), excluding nine cases that reported ‘multiple’. A histologic diagnosis was rendered in 100% of CNB procedures (diagnostic yield 100%), with the most frequent diagnoses being tenosynovial giant cell tumour (TGCT) at 30% (Figure 1), ganglion cyst at 11% (Figure 2), peripheral neural sheath tumour (neurofibroma), haemangioma and fibrofatty tissue each at 8%. The frequency of epidermal inclusion cyst was at 5%. CNB was 100% sensitive in detecting the three (8%) malignancies: myxoinflammatory fibroblastic sarcoma (recurrent), carcinosarcoma (Figure 3) and metastatic squamous cell carcinoma (Table 1).

**Table 1:** Initial core‐needle biopsy results.

Benign	Number (n = 34)	Malignant	Number (n = 3)
Palmar fibromatosis	2	Myxoinflammatory fibroblastic sarcoma, grade 2/3	1
PNST	3	Carcinosarcoma	1
Angioleiomyoma	1	Metastatic squamous cell carcinoma	1
Benign cyst^a^	6		
TGCT	11		
Granuloma	1		
Haemangioma	3		
Fibrofatty tissue	3		
Lipoma	2		
Necrotic tissue	1		
Benign fibrous histiocytoma	1		

PNST, peripheral nerve sheath tumour; TGCT, tenosynovial giant cell tumour.

^a^ Four ganglion cysts and two epidermal inclusion cysts.

Of the 37 tumours that were biopsied, 16 were surgically excised (Table 2). There was only one discrepancy between biopsy and excisional pathology. One excised angiomyoma was initially diagnosed as haemangioma on CNB, but it did not alter management. Multiple CNBs were obtained for both discordant results. All other histologic results were concordant for a diagnostic accuracy of 97%. There was a mean interval of 112 days between CNB and surgical excision and specifically a mean interval of 29 days between CNB and surgical excision for the three malignancies. Only two minor CNB complications occurred, one of which was post‐procedural paraesthesia from a TGCT encasing the digital nerve, which has diminished over time but is still ongoing. The other complication was post‐biopsy bruising from palmar fibromatosis, which has since resolved. Both complications were considered Grade A according to the SIR guidelines.

**Table 2:** Final excisional histopathologic results.

Benign	Number (n = 13)	Malignant	Number (n = 3)
Palmar fibromatosis	1	Myxoinflammatory fibroblastic sarcoma, grade 2/3	1
PNST	2	Carcinosarcoma	1
Angiomyoma	1	Metastatic squamous cell carcinoma	1
Benign cyst^a^	2		
TGCT	6		
Granuloma	1		

PNST, peripheral nerve sheath tumour; TGCT, tenosynovial giant cell tumour.

^a^ One epidermal inclusion cyst and one pilar cyst.

## Discussion

Current studies report the diagnostic yield of CNB in musculoskeletal lesions ranging from 77 to 90%.^7^, ^8^, ^9^ Our study demonstrated CNB in the hands and fingers to have a diagnostic yield of 100%, and a diagnostic accuracy of 97%, comparable or even superior to previous findings. Additionally, there were no serious post‐procedural complications after CNB, and minor complications were rare. These observations accord with a meta‐analysis that reported a significantly reduced complication rate in CNB patients of 1% compared to 4% in patients who underwent excisional biopsy for soft tissue sarcoma.^3^

In our study, we found that 2 of 37 patients (5%) experienced minor complications. These included post‐procedural paraesthesia after CNB of a TGCT encasing the digital nerve, and bruising post‐CNB of palmar fibromatosis. Tenosynovial giant cell tumours have been reported to involve peripheral nerves, and patients may experience paraesthesia as a result.^10^, ^11^ Currently, surgical resection remains the treatment of choice for patients with TGCT.^12^ However, patients with unresectable disease, as in the case of infiltrative tumour growth, may benefit from systemic therapy using CSF‐1R inhibitors by avoiding surgery and helping improve functional outcomes.^12^ In cases where patients do not experience peripheral nerve deficit prior to biopsy, the possibility of nerve disruption during CNB should be considered. The other complication of bruising following CNB was minor and self‐limited. We believe the minor risks inherent in the procedure should not deter image‐guided CNB of hand and finger soft tissue masses.

Our study found a 100% diagnostic yield of CNB in the hand and fingers, higher than other reports of CNB of soft tissue masses.^7^, ^8^, ^9^ Reasons for the one discrepant biopsy result in this population include heterogeneity of tumour tissue and small tumour sizes, leading to sampling error.^13^ Notably, the discrepancy was not considered clinically meaningful because there was no change in patient management for this benign lesion. Image guidance using high‐frequency probes enables direct real‐time visualisation of needle passes through the lesion, which increases diagnostic yield.^3^, ^9^ A 16G needle was used for this discrepant case and ‘multiple’ core needle biopsies were obtained. In our study, 18‐gauge needles were most frequently used. No biopsies were performed using needles larger than 14 gauge, as they provide little to no incremental gain in diagnostic yield.^3^

In one of our cases, an angioma was diagnosed using CNB as a haemangioma using a 16‐gauge needle. While this misdiagnosis did not alter treatment management, it is interesting that ultrasound‐guided CNB did not yield accurate results as the lesion measured 4.3 cm in diameter.

The use of MRI prior to biopsy should be explored to provide a more comprehensive picture of tumour characteristics before definitive treatment of soft tissue masses of the hand. It would be valuable to investigate the role of pre‐procedural MRI, particularly in cases where there is a discrepancy between ultrasound‐guided CNB and final pathology reports to guide surgical planning and determine the need for further biopsy.

Even though ganglion cysts are frequently encountered and do not routinely require histologic confirmation, some were included in our study because of equivocal imaging features, such as complex thickened walls that might simulate more solid components of a cystic neoplasm. We included these cases since the procedural risks of injury to adjacent vessels, nerves and tendons are similar to those incurred for biopsy of solid masses.

Previous studies have stated that lesions larger than 1 cm can reliably be sampled without compromising diagnostic yield.^7^ Interestingly, our study demonstrated successful diagnostic yield of all six lesions <1 cm. This is notable given the compact architecture of the hand and proximity to numerous tendons and neurovascular bundles that render these biopsies technically challenging. Accordingly, we advocate meticulous procedural technique with visualisation of the biopsy needle at all times utilising an in‐plane approach, to minimise the risk of inadvertent tendon, nerve injury or vascular puncture. Additionally, following orthopaedic oncology principles, biopsy approaches were selected to avoid transgression of more than a single compartment.

The fact that needle gauge did not seem to influence diagnostic yield is a somewhat counterintuitive finding, since thicker cores might be presumed to better retain tissue architecture during specimen processing and contain diagnostic tissue. However, the failure to correlate needle gauges with diagnostic yield has been observed in other studies.^9^, ^14^ We suspect several factors account for the overall high diagnostic yield regardless of needle gauge, including (i) direct visualisation of the specimen chamber within the targeted lesion, ensuring adequate tissue sampling; (ii) review of histology by an expert bone and soft tissue pathologist with 35+ years of subspecialty experience; and (iii) availability of immunohistochemical stains that enhance diagnostic confidence even with scant tissue. Ultimately, this sample size is admittedly underpowered to detect a small difference in diagnostic yield between needle gauges, but our results support the prudent use of smaller gauges to minimise procedural risk.

Among the limitations of this study are that it is retrospective in nature. There was no standard protocol dictating circumstances for choosing gauge or number of core samples among all lesions. Variations in tumour characteristics and local anatomy guide needle size and number of samples taken, both of which were determined by the radiologist at the time of the procedure. Similarly, the decision on whether or not to biopsy a lesion is physician dependent and is not standardised at our institution. Some characteristics of soft tissue masses and some masses have distinct radiologic and clinical characteristics that may obviate the need for biopsy. Indications for biopsy generally included those masses with indeterminate imaging findings, or where definitive tissue diagnosis was required to guide appropriate therapy: for example, biopsy of a complex cyst with a thickened wall mimicking the solid component of a cystic neoplasm, or biopsy to confirm suspected TGCT to enable treatment with pexidartinib. Surgeons involved in the care of these patients were either fellowship‐trained musculoskeletal oncology surgeons or hand surgeons with experience in hand tumours. Length of clinical practice ranged from 3 to 20+ years of clinical practice. Additionally, as a sarcoma centre, we receive referrals in which diagnosis is known at the time of biopsy. Thus, our population may observe a larger prevalence of malignancies than the general population, and our findings may overestimate the predictive value of ultrasound‐guided CNB. Keeping this in mind, this study is useful to raise awareness of potentially malignant cases that should not be underestimated in the daily clinical practice of community surgeons. It is important to note that our study did not evaluate the accuracy and complication rate of non‐image‐guided CNB, which may carry higher risks of complications than are observed in this cohort. Another limitation of our study is that the size of needles and number of passes were not recorded in all cases, but this would not fundamentally change our conclusions.

Overall, ultrasound‐guided CNB is safe and accurate in diagnosing soft tissue tumours of the hand and fingers, enabling definitive treatment planning with more conservative utilisation of OR resources. Interestingly, diagnostic yield was not adversely affected by needle gauge and appears to be unrelated to the number of passes made (although we advocate a minimum of two). Lesion size also had little effect on diagnostic yield, with masses as small as 4 mm successfully diagnosed by CNB. We observed that increased lesion diameter did not always correlate with increased diagnostic accuracy in the hand, a finding that should be confirmed by examining a larger patient population.

## Author Contributions

**Stephanie Magoon:** Data curation (lead); formal analysis (lead); investigation (lead); methodology (lead); project administration (lead); visualization (lead); writing – original draft (lead); writing – review and editing (lead). **Vanessa Peters:** Visualization (supporting); writing – original draft (supporting); writing – review and editing (supporting). **Felipe Ferreira de Souza:** Conceptualization (equal); formal analysis (equal); writing – review and editing (equal). **David Chen:** Conceptualization (equal); formal analysis (equal); writing – review and editing (equal). **Patrick Owens:** Conceptualization (equal); formal analysis (equal); writing – review and editing (equal). **Juan Pretell‐Mazzini:** Conceptualization (equal); formal analysis (equal); writing – review and editing (supporting). **Natalia Fullerton:** Conceptualization (equal); formal analysis (equal); writing – review and editing (equal). **Jean Jose:** Conceptualization (equal); formal analysis (equal); writing – review and editing (equal). **Andrew Rosenberg:** Conceptualization (equal); formal analysis (equal); writing – review and editing (equal). **Ty K. Subhawong:** Conceptualization (lead); supervision (lead); writing – review and editing (supporting).
